# Supplementary material for: Vibrational Spectroscopy Combined with Chemometrics as Tool for Discriminating Organic vs. Conventional Culture Systems for Red Grape Extracts
Source: Foods. 2021 Aug 11;10(8):1856. doi: 10.3390/foods10081856 (PMC8393556; doi:10.3390/foods10081856)
Supplement: Supplementary file 1 [file foods-10-01856-s001.zip › foods-1284896-SI.pdf]

foods

MDPI

Article

Vibrational Spectroscopy Combined with Chemometrics as Tool for Discriminating Organic vs. Conventional Culture Systems for Red Grape Extracts

Cristiana Radulescu [1,2], Radu Lucian Olteanu [2,*], Cristina Mihaela Nicolescu [2], Marius Bumbac [1,2], Lavinia Claudia Buruleanu [3] and Georgeta Carmen Holban [4]

1 Faculty of Sciences and Arts, Valahia University of Targoviste, 130004 Targoviste, Romania; cristiana.radulescu@valahia.ro (C.R.); marius.bumbac@valahia.ro (M.B.)
2 Institute of Multidisciplinary Research for Science and Technology, Valahia University of Targoviste, 130004 Targoviste, Romania; cristina.nicolescu@valahia.ro
3 Faculty of Environmental Engineering and Food Science, Valahia University of Targoviste, 130004 Targoviste, Romania; lavinia.buruleanu@valahia.ro
4 Doctoral School, University of Agronomic Sciences and Veterinary Medicine of Bucharest, 011464 Bucharest, Romania; carmenholban@yahoo.com
* Correspondence: radu.olteanu@valahia.ro

Abstract: Food plants provide a regulated source of delivery of functional compounds, plant secondary metabolites production being also tissue specific. In the grape berries, the phenolic compounds, flavonoid and non-flavonoids, are distributed in the different parts of the fruit. The aim of this study was to investigate the applicability of FTIR, and Raman screening spectroscopic techniques combined with multivariate statistical tools to find patterns in red grapes berry parts (skin, seeds and pulp) according to grape variety and vineyard type (organic and conventional). Exploratory data analysis has revealed hidden patterns in complex spectral data by reducing the information to a more comprehensible form and indicating whether there are patterns or trends in the data. Spectral data were acquired and processed using same pattern for each different berry parts (skin, seeds and pulp). Vibrational spectroscopic techniques, attenuated total reflectance-Fourier transform infrared (ATR-FTIR) and Raman, were proven useful in differentiation of the extracts as it provided information on the vibrational bands which are related to the chemical composition. Multivariate analysis has allowed a separation between extracts obtained from organic and conventional vineyards for each grape variety for all grape berry parts. A well-defined differentiation based on red grape variety could not be highlighted.

Keywords: vibrational spectroscopy; red grapes extracts; organic/conventional vineyards; chemometrics

Citation: Radulescu, C.; Olteanu, R.L.; Nicolescu, C.M.; Bumbac, M.; Buruleanu, L.C.; Holban, G.C. Vibrational Spectroscopy Combined with Chemometrics as Tool for Discriminating Organic vs. Conventional Culture Systems for Red Grape Extracts. *Foods* **2021**, *10*, 1856. https://doi.org/10.3390/foods10081856

Academic Editor: Francesco Longobardi, Ana María Jiménez Carvelo and Antonello Santini

Received: 18 June 2021
Accepted: 7 August 2021
Published: 11 August 2021

Publisher's Note: MDPI stays neutral with regard to jurisdictional claims in published maps and institutional affiliations.

Copyright: © 2021 by the authors. Submitted for possible open access publication under the terms and conditions of the Creative Commons Attribution (CC BY) license (http://creativecommons.org/licenses/by/4.0/).

Supplementary

The acquired FTIR spectral data (transmittance mode) were arranged in the form of an 8x1866 matrix corresponding to organic and conventional extracts (observations/samples) and transmittance readings per spectrum (variables), considering the spectral region 4000-400 cm^{-1}; similarly, Raman spectral data were arranged in an 8x223 matrix using 2000-200 cm^{-1} spectral region. After correction for the background spectrum, all analyzed spectra showed different weak, medium and strong vibrational frequencies; in spectroscopic terms the weak, medium and strong peaks (intensity), together with wavenumbers, allow the assignment of functional groups from organic compounds. The ATR correction algorithm can 'symmetrize' the peak shape; when the second derivative is plotted out, the peak positions are seen at the different wavenumbers (as a week, me-

dium or strong peaks) [43]. The background spectrum can be defined as a single beam spectrum acquired with no sample in the infrared beam [44]. The purpose of a background spectrum is to measure the contribution of the instrument and environment to the spectrum. These effects are removed from a sample spectrum by rationing the sample single beam spectrum to the background spectrum [38]. Spectral data pre-processing is a first step in the workflow of infrared (IR) and Raman spectra analysis which involves specific processing procedures performed on the raw data. Pre-processing has been shown to be significant for subsequent data mining tasks; it is now widely recognized that quantitative and classification models developed on the basis of pre-processed data generally perform better than models that solely use raw data [41,45,46]. In transmission type IR spectroscopy, spectral baselines can be distorted as a result of scattering, absorption by the supporting substrate, changing conditions during data collection, or the variableness due to instrumental factors. Subtracting the estimation of a background from the un-processed spectrum leads to a more interpretable signal, allowing to determine spectral parameters (band positions, intensity values) more accurately [47]. On all FTIR spectra corresponding to the investigated samples extracts baseline correction, using scattering correction method (10 iterations, 64 baseline points), was applied during spectral data acquisition. In the case of Raman spectra for investigated extracts, spectral data acquisition baseline correction function is built in, which greatly facilities reduction in fluorescence interference and accounts for drift in background. Baseline constructed from a multi-segment, smoothed polynomial allows effective removal of fluorescence background and slanted baseline with minimal artificial bias. The use of IR spectra in classification analysis typically requires some form of normalization that allows an effective comparison across heterogeneous sets of samples [42,48]. Normalization has been thus identified as one important pre-processing method which is commonly applied to minimize the effects of varying optical pathlengths on the data, or to compensate for intensity variations of the source (e.g., IR synchrotron) to mention one of the possible instrumental causes [41]. The result of normalization is a spectrum which is scaled, and offset corrected at the same time [41]. Box-Cox transformation [49-51] was applied before statistical analysis of the spectral data to obtain approximately normally distributed values.

For outlier detection multidimensional tests were applied as one of the most common or standard method is based on Mahalanobis distance; the sensitivity of this method in detecting outliers depends on the conditions of how different the mean of the outliers is relative to the remainder of the sample and the different covariance matrix structures [39]. The tests implemented are used to compare samples described by several variables: Mahalanobis distance (the Bonferroni correction was used for the alpha significance level set to 5%), Wilk's lambda (Rao's approximation), and Box and Kullback's tests (for testing the equality of the within-groups covariance matrices); the tests were performed assigning two groups (four extracts in each group) based on vineyard type (Table 1). Tests on averages identify the difference: the test of Wilks' lambda concludes that there is a significant difference between the groups' means (at least one of the means vector is different from another, as the computed p value was lower than the significance level alpha).

Principal Component Analysis (PCA) allows the visualization of the information in the large data set in a few principal components while retaining the maximum possible variability within that set. Principal component analysis was used to reduce the dimensionality of the spectral data to a smaller number of components, facilitating the subsequent analysis. PCA was performed on the FTIR and Raman spectra of the extract samples, separately, to examine the possible grouping of samples related to grape varieties and vineyard type. The procedure transforms a number of possibly correlated variables into a smaller number of uncorrelated variables called principal components [35,38,52-54]; the principal components are linear combinations of the original variables weighted by their contribution to explaining the variance in a particular orthogonal di-

mension; the new axes allow the investigation of data matrices with many variables and the display of the multivariate nature in a relatively small number of dimensions [55-57].

Agglomerative Hierarchical Clustering (AHC) was performed using the Euclidean distance as the distance measure and single linkage strategy to link clusters within the data set (Ward's method). AHC orders a large complex data which are graphically displayed in a dendrogram which shows the level of similarity between individual sample and groups of samples relative to the entire dataset. The dendrogram is produced typically using agglomerative methods where clustering starts with individual sample and proceeds sequentially until all samples are linked together to form clusters [58,59]. It was applied automatic truncation so that the results show the groups to which observation belongs; automatic truncation is based on entropy and tries to create homogenous groups [38]. When the increase in dissimilarity level is strong, it has reached a level where the grouping implies groups that are already homogenous; automatic truncation uses this criterion to decide when to stop aggregating observations (or groups of observations). In AHC the data are not partitioned into a particular cluster in a single step. Instead of this, a series of partitions takes place, which may run from a single cluster containing all objects to n clusters each, containing a single object. In order to build up these groups a measurement of the similarity between the different objects is considered (this measurement is also known as the distance between the objects considered) [52]. The AHC used under the software (XLSTAT) uses dissimilarities; the conversion for each object pair consists in taking the maximum similarity for all pairs and subtracting from this the similarity of the pair in question. Among all the linkage cluster methods, in this study, the agglomerative Ward's method has been selected who generate the different clusters in order to minimize the loss associated with each cluster [38].

Discriminant Analysis (DA) is a statistical method that can be used in explanatory or predictive frameworks. Two models of DA are used depending on a basic assumption: if the covariance matrices are assumed to be identical, linear discriminant analysis is used. If, on the contrary, it is assumed that the covariance matrices differ in at least two groups, then the quadratic discriminant analysis should be preferred. The Box test is used to test this hypothesis (the Bartlett approximation enables a Chi2 distribution to be used for the test). It is common to start with linear analysis then, depending on the results from the Box test, to carry out quadratic analysis if required. As for linear and logistic regression, efficient stepwise methods have been proposed. They can, however, only be used when quantitative variables are selected as the input and output tests on the variables assume that are normally distributed. The stepwise method gives a more appropriate model which avoids variables which contribute only little to the model [52,60]. When the number of variables exceeds the number of samples, one method of multivariate discrimination is to use principal components analysis to reduce the dimensionality and then to perform canonical variates analysis [61,62].

The partial bootstrap method consists in drawing a number of samples (with replacement) each of the same size as the matrix of data used for the PCA. Then, each sample is centered, and normalized in the case of normalized PCA, and each observation of each sample is displayed on the PC plans as supplementary observation. As a consequence, a cloud of bootstrap observations is generated around each original observation.

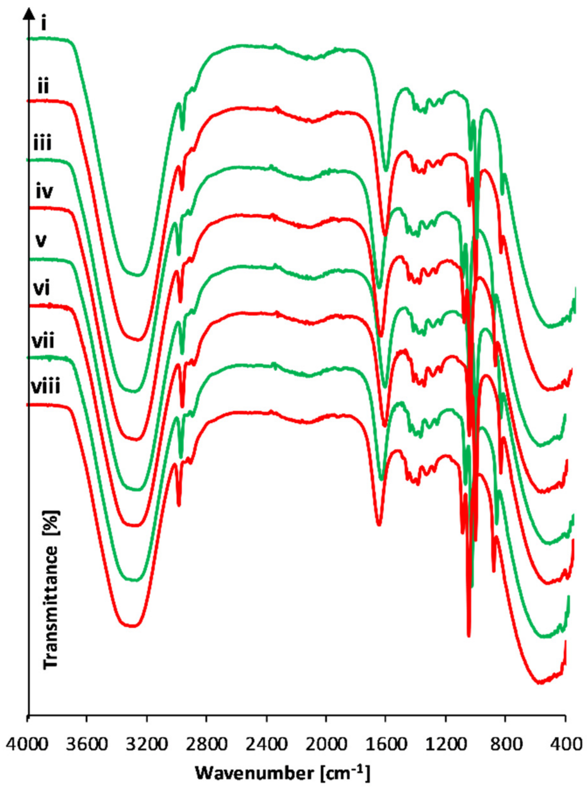

Figure S1. Overlaps of Fourier transform infrared (FTIR) spectra for red grapes skin extracts: i) M-O, ii) M-C, iii) FN-O, iv) FN-C, v) PN-O, vi) PN-C, vii) MH-O, viii) MH-C.

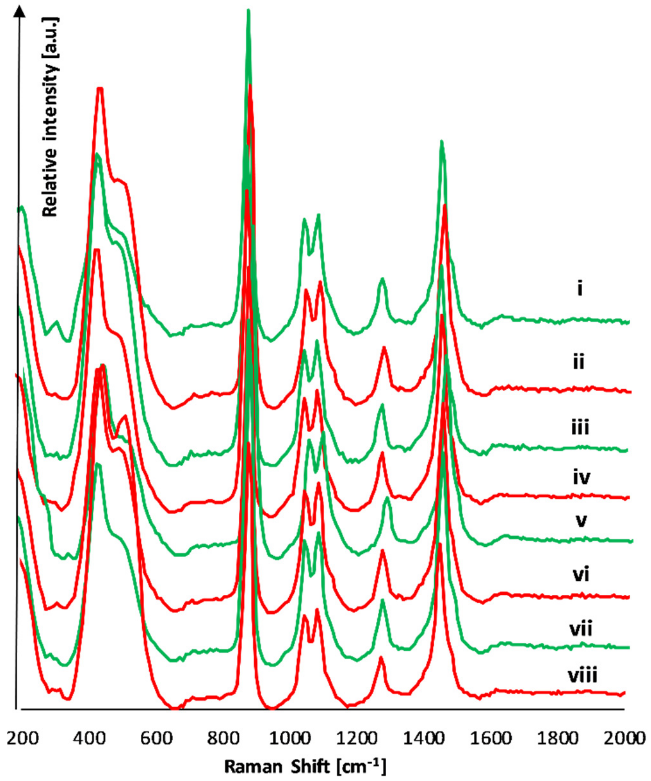

Figure S2. Overlaps of Raman spectra for red grapes skin extracts: i) M-O, ii) M-C, iii) FN-O, iv) FN-C, v) PN-O, vi) PN-C, vii) MH-O, viii) MH-C.

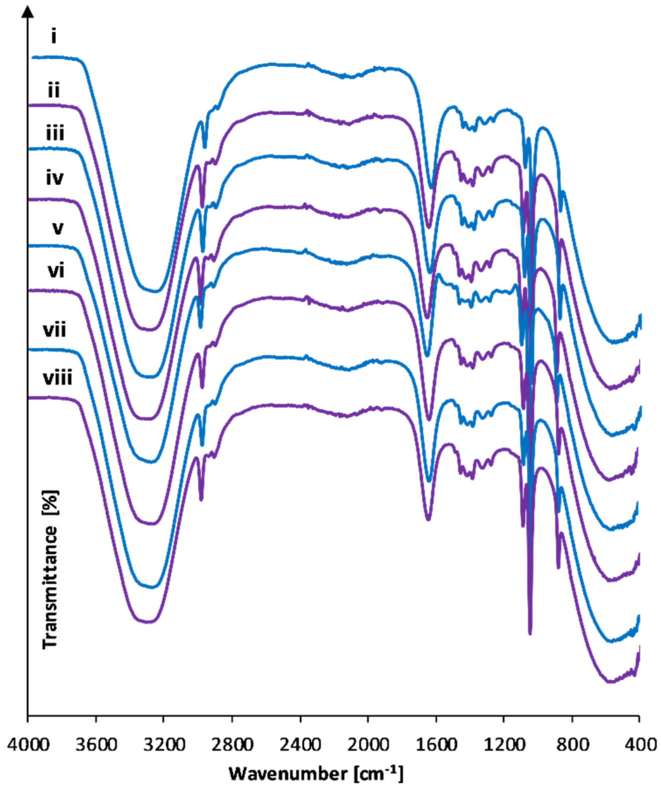

Figure S3. Overlaps of Fourier transform infrared (FTIR) spectra for red grapes seeds extracts: i) M-O, ii) M-C, iii) FN-O, iv) FN-C, v) PN-O, vi) PN-C, vii) MH-O, viii) MH-C.

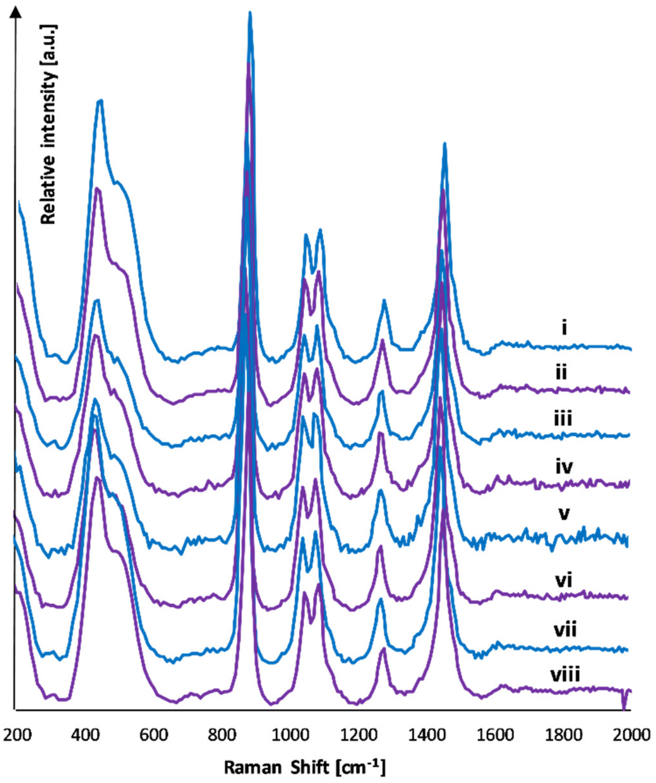

Figure S4. Overlaps of Raman spectra for red grapes seeds extracts: i) M-O, ii) M-C, iii) FN-O, iv) FN-C, v) PN-O, vi) PN-C, vii) MH-O, viii) MH-C.

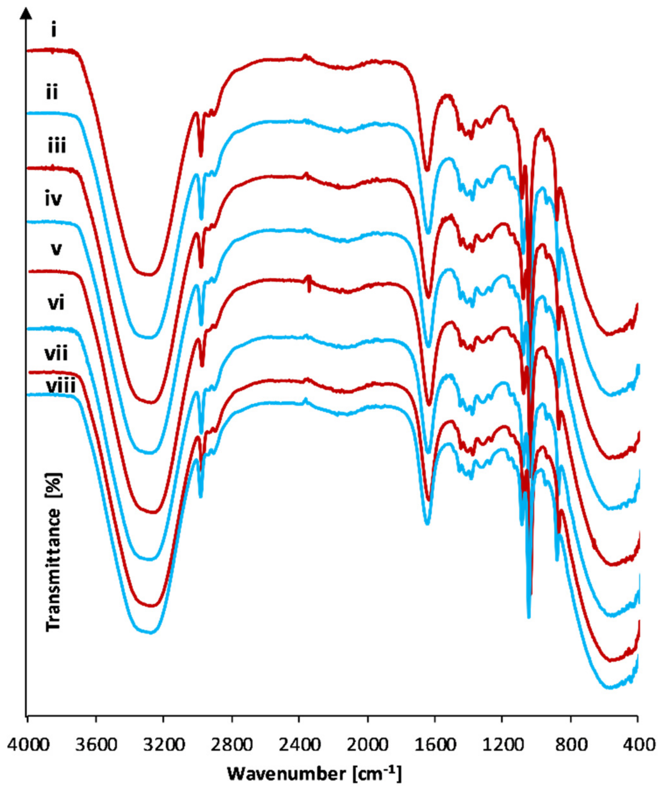

Figure S5. Overlaps of Fourier transform infrared (FTIR) spectra for red grapes pulp extracts: i) M-O, ii) M-C, iii) FN-O, iv) FN-C, v) PN-O, vi) PN-C, vii) MH-O, viii) MH-C.

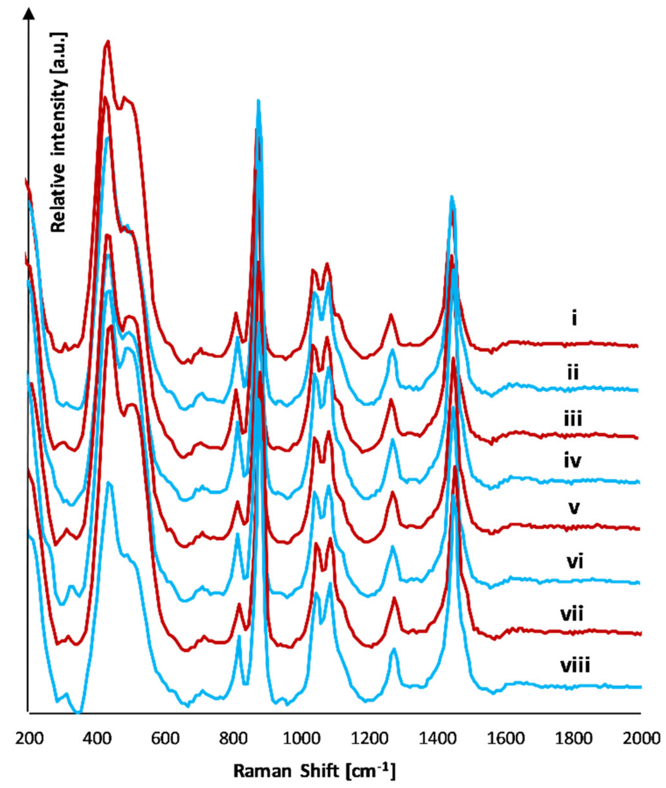

Figure S6. Overlaps of Raman spectra for red grapes pulp extracts: i) M-O, ii) M-C, iii) FN-O, iv) FN-C, v) PN-O, vi) PN-C, vii) MH-O, viii) MH-C.

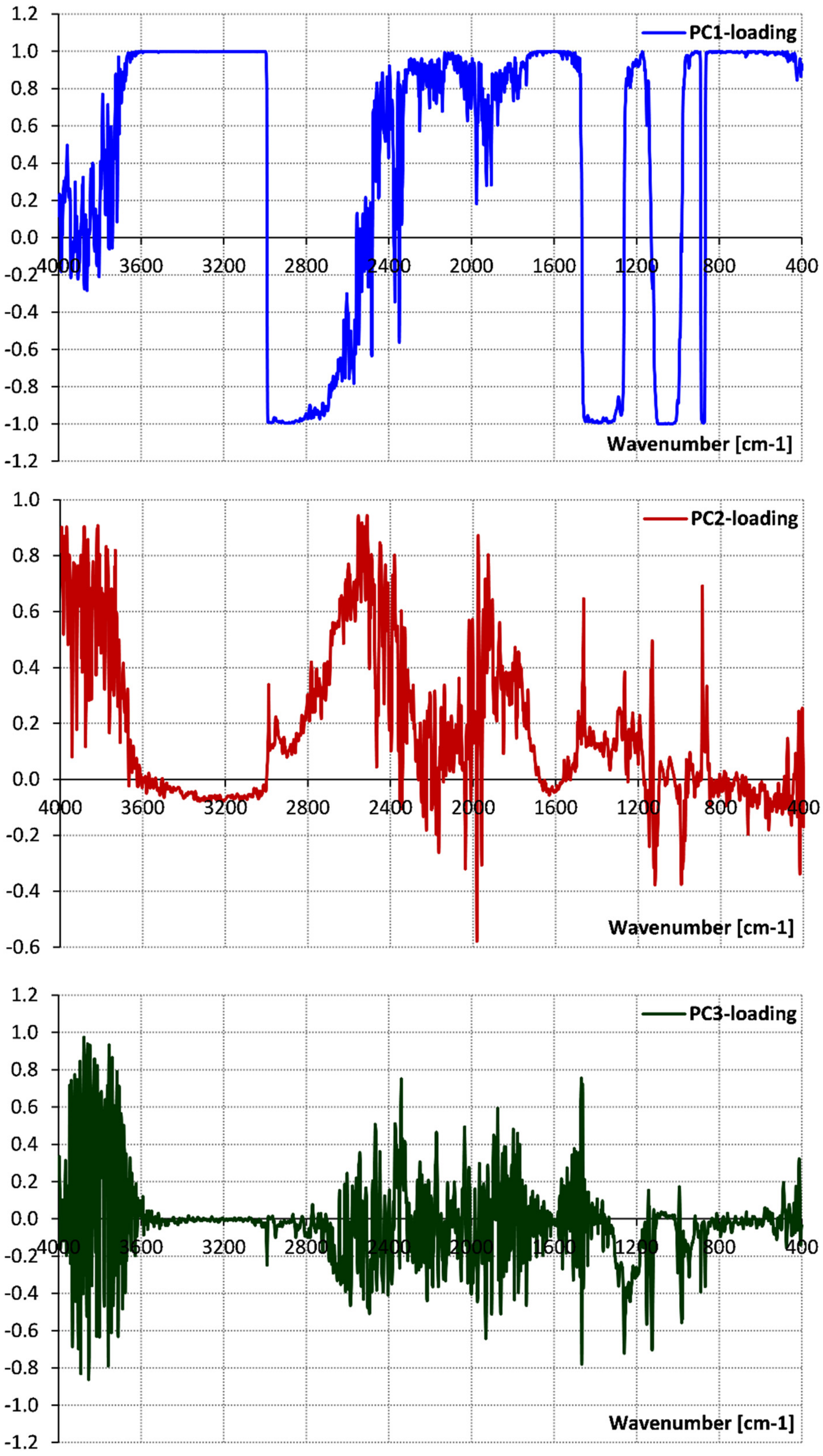

Figure S7. Principal components (PCs) loadings for the first three PCs derived from FTIR spectral data of the red grape skin extracts.

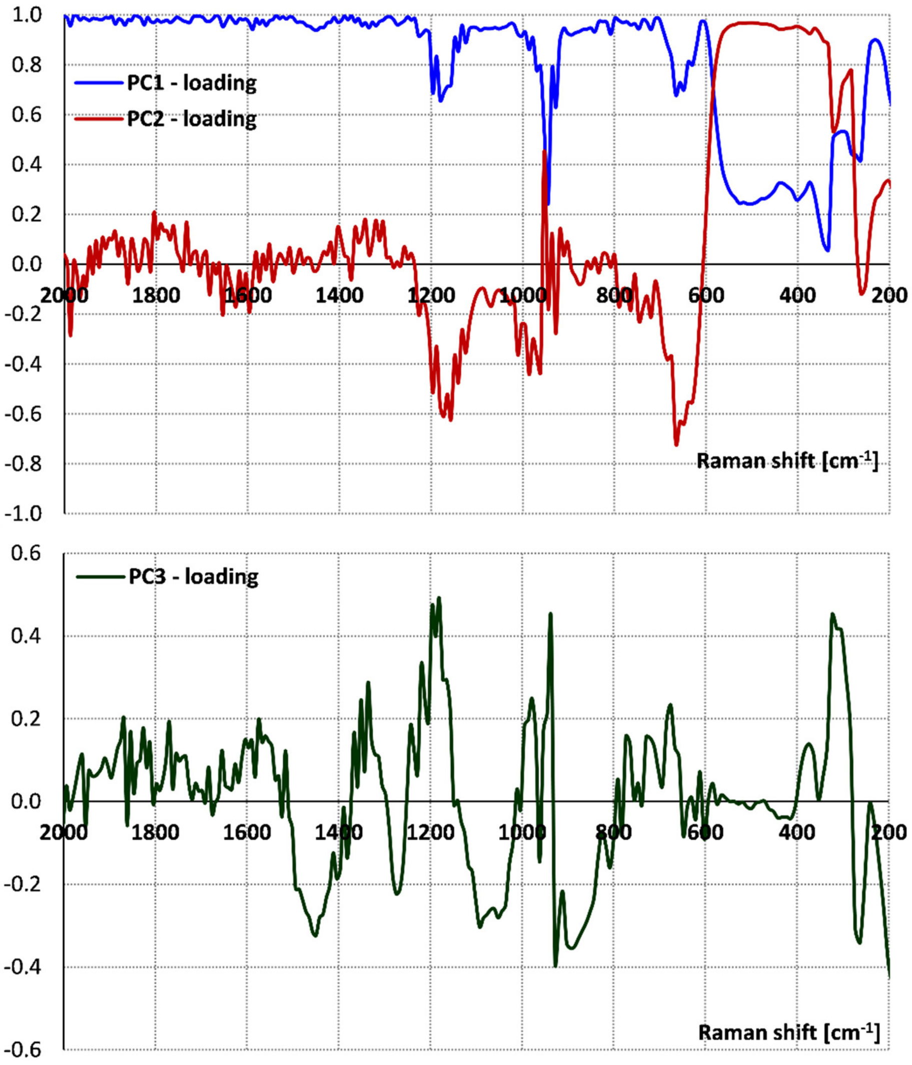

Figure S8. Principal components (PCs) loadings for the first three PCs derived from Raman spectral data of the red grape skin extracts.

Table S1. Confusion matrix for the training sample (FTIR data – red grape skin extracts).

From / to	Conventional	Organic	Total	% correct
Conventional	4	0	4	100.00
Organic	1	3	4	75.00
Total	5	3	8	87.50

Table S2. Cross-validation (FTIR data – red grape skin extracts): prior and posterior classification and membership probabilities.

Extract	Prior	Posterior	Conventional	Organic
M-O	Organic	Organic	0.000	1.000
FN-O	Organic	Organic	0.000	1.000
PN-O	Organic	Organic	0.483	0.517
MH-O	Organic	Organic	0.000	1.000
M-C	Conventional	Conventional	0.999	0.001
FN-C	Conventional	Conventional	0.917	0.083
PN-C	Conventional	Conventional	1.000	0.000
MH-C	Conventional	Conventional	0.999	0.001

Table S3. Confusion matrix for the cross-validation results (FTIR data – red grape skin extracts).

From / to	Conventional	Organic	Total	% correct
Conventional	4	0	4	100.00
Organic	0	4	4	100.00
Total	4	4	8	100.00

Table S4. Confusion matrix for the training sample (Raman data – red grape skin extracts).

From / to	Conventional	Organic	Total	% correct
Conventional	4	0	4	100.00
Organic	0	4	4	100.00
Total	4	4	8	100.00

Table S5. Cross-validation (Raman data – red grape skin extracts): prior and posterior classification and membership probabilities.

Extract	Prior	Posterior	Conventional	Organic
M-O	Organic	Organic	0.002	0.998
FN-O	Organic	Organic	0.000	1.000
PN-O	Organic	Organic	0.033	0.967
MH-O	Organic	Organic	0.028	0.972
M-C	Conventional	Conventional	1.000	0.000
FN-C	Conventional	Conventional	1.000	0.000
PN-C	Conventional	Conventional	1.000	0.000
MH-C	Conventional	Conventional	1.000	0.000

Table S6. Confusion matrix for the cross-validation results (Raman data – red grape skin extracts).

From / to	Conventional	Organic	Total	% correct
Conventional	4	0	4	100.00
Organic	0	4	4	100.00
Total	4	4	8	100.00

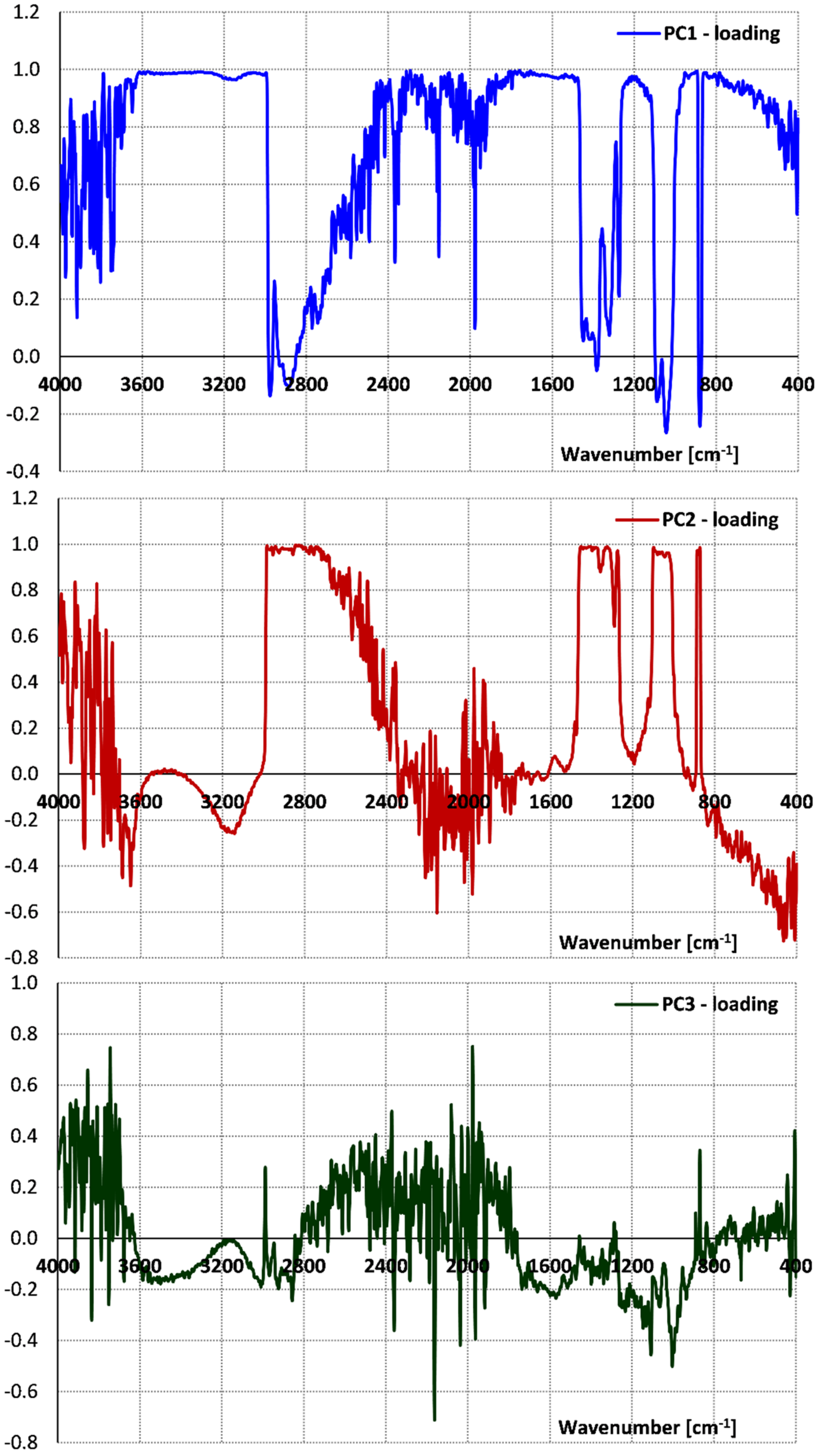

Figure S9. Principal components (PCs) loadings for the first three PCs derived from FTIR spectral data of the red grape seeds extracts.

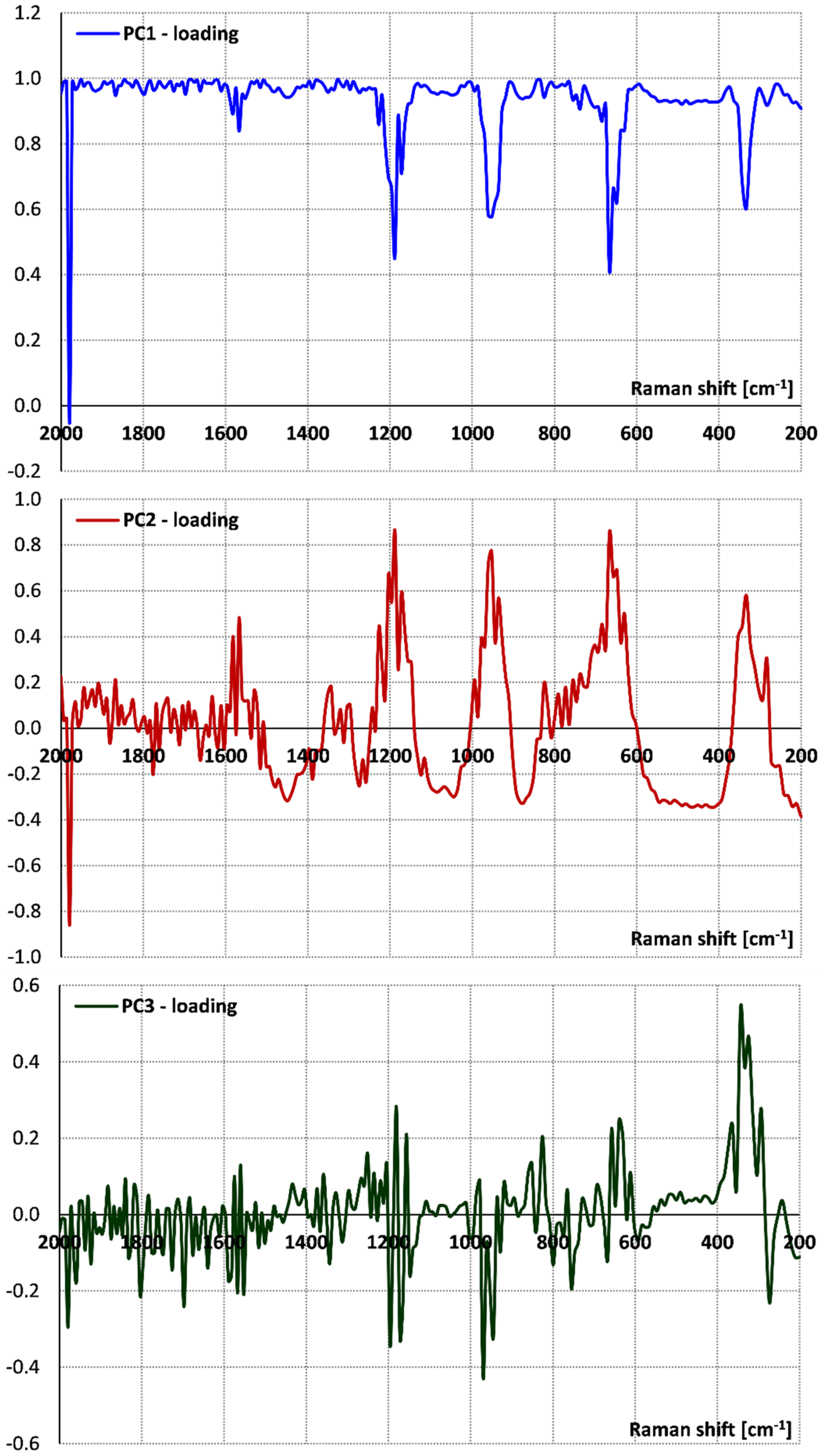

Figure S10. Principal components (PCs) loadings for the first three PCs derived from Raman spectral data of the red grape seeds extracts.

Table S7. Confusion matrix for the training sample (FTIR data – red grape seeds extracts).

From / to	Conventional	Organic	Total	% correct
Conventional	4	0	4	100.00
Organic	0	4	4	100.00
Total	4	4	8	100.00

Table S8. Cross-validation (FTIR data – red grape seeds extracts): prior and posterior classification and membership probabilities.

Extract	Prior	Posterior	Conventional	Organic
M-O	Organic	Organic	0.000	1.000
FN-O	Organic	Organic	0.000	1.000
PN-O	Organic	Organic	0.000	1.000
MH-O	Organic	Organic	0.000	1.000
M-C	Conventional	Conventional	1.000	0.000
FN-C	Conventional	Conventional	1.000	0.000
PN-C	Conventional	Conventional	0.955	0.045
MH-C	Conventional	Conventional	1.000	0.000

Table S9. Confusion matrix for the cross-validation results (FTIR data – red grape seeds extracts).

From / to	Conventional	Organic	Total	% correct
Conventional	4	0	4	100.00
Organic	0	4	4	100.00
Total	4	4	8	100.00

Table S10. Confusion matrix for the training sample (Raman data – red grape seeds extracts).

From / to	Conventional	Organic	Total	% correct
Conventional	4	0	4	100.00
Organic	0	4	4	100.00
Total	4	4	8	100.00

Table S11. Cross-validation (Raman data – red grape seeds extracts): prior and posterior classification and membership probabilities.

Extract	Prior	Posterior	Conventional	Organic
M-O	Organic	Organic	0.000	1.000
FN-O	Organic	Organic	0.000	1.000
PN-O	Organic	Organic	0.000	1.000
MH-O	Organic	Organic	0.000	1.000
M-C	Conventional	Conventional	0.990	0.010
FN-C	Conventional	Conventional	0.994	0.006
PN-C	Conventional	Conventional	0.910	0.090
MH-C	Conventional	Conventional	1.000	0.000

Table S12. Confusion matrix for the cross-validation results (Raman data – red grape seeds extracts).

From / to	Conventional	Organic	Total	% correct
Conventional	4	0	4	100.00
Organic	0	4	4	100.00
Total	4	4	8	100.00

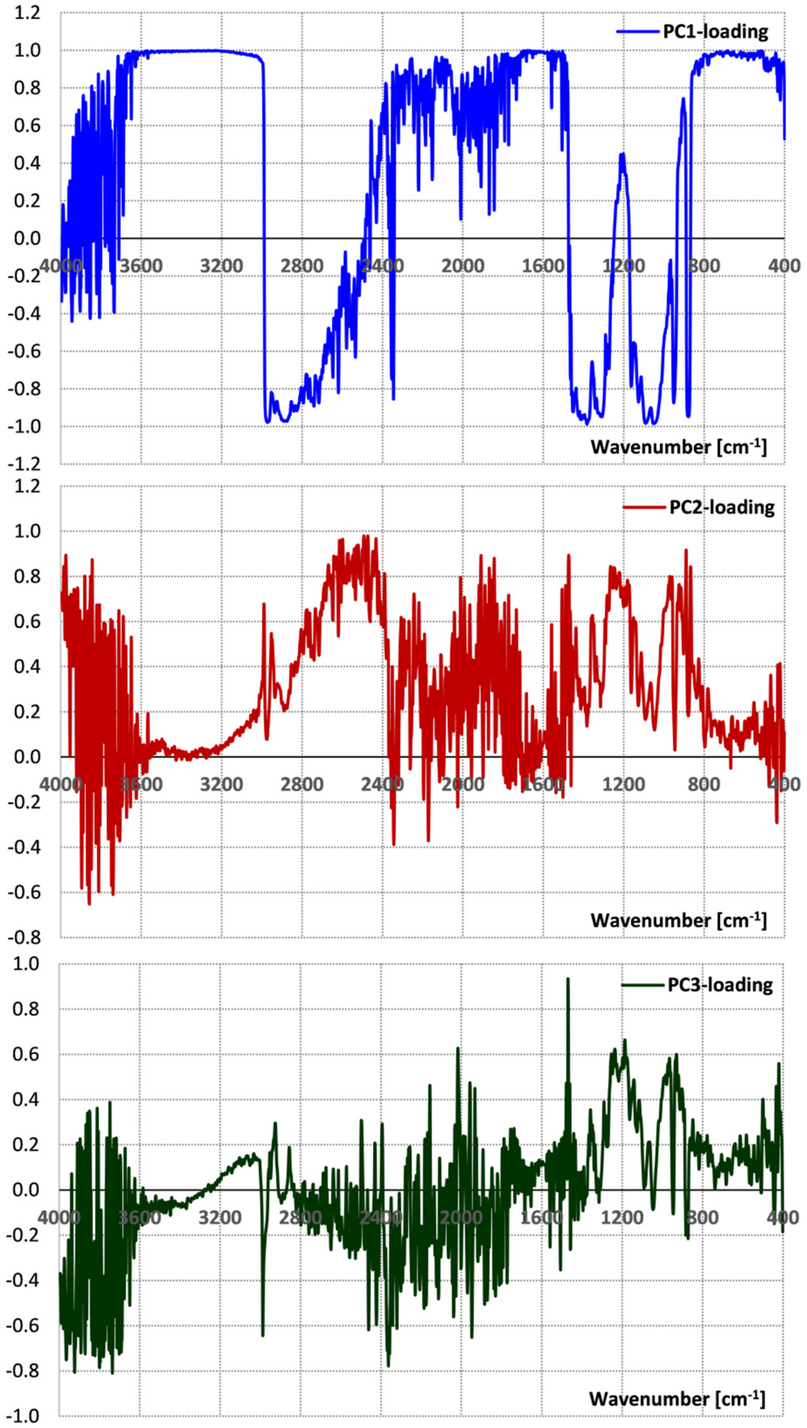

Figure S11. Principal components (PCs) loadings for the first three PCs derived from FTIR spectral data of the red grape pulp extracts.

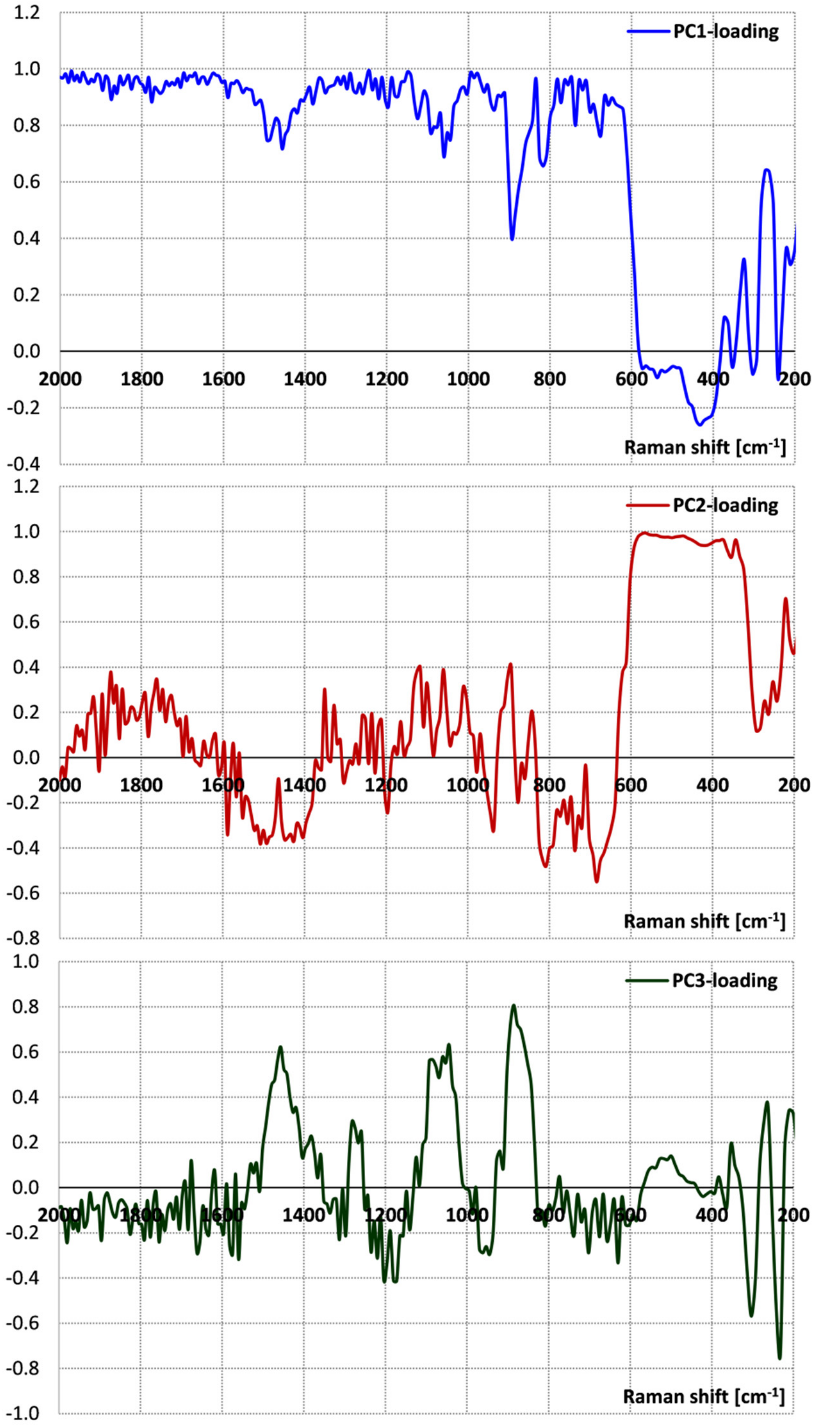

Figure S12. Principal components (PCs) loadings for the first three PCs derived from Raman spectral data of the red grape pulp extracts.

Table S13. Confusion matrix for the training sample (FTIR data – red grape pulp extracts).

From / to	Conventional	Organic	Total	% correct
Conventional	4	0	4	100.00
Organic	0	4	4	100.00
Total	4	4	8	100.00

Table S14. Cross-validation (FTIR data – red grape pulp extracts): prior and posterior classification and membership probabilities.

Extract	Prior	Posterior	Conventional	Organic
M-O	Organic	Organic	0.000	1.000
FN-O	Organic	Organic	0.000	1.000
PN-O	Organic	Organic	0.000	1.000
MH-O	Organic	Organic	0.000	1.000
M-C	Conventional	Conventional	1.000	0.000
FN-C	Conventional	Conventional	0.992	0.008
PN-C	Conventional	Conventional	0.982	0.018
MH-C	Conventional	Conventional	0.998	0.002

Table S15. Confusion matrix for the cross-validation results (FTIR data – red grape pulp extracts).

From / to	Conventional	Organic	Total	% correct
Conventional	4	0	4	100.00
Organic	0	4	4	100.00
Total	4	4	8	100.00

Table S16. Confusion matrix for the training sample (Raman data – red grape pulp extracts).

From / to	Conventional	Organic	Total	% correct
Conventional	4	0	4	100.00
Organic	0	4	4	100.00
Total	4	4	8	100.00

Table S17. Cross-validation (Raman data – red grape pulp extracts): prior and posterior classification and membership probabilities.

Extract	Prior	Posterior	Conventional	Organic
M-O	Organic	Organic	0.000	1.000
FN-O	Organic	Organic	0.002	0.998
PN-O	Organic	Organic	0.043	0.957
MH-O	Organic	Organic	0.035	0.965
M-C	Conventional	Conventional	0.746	0.254
FN-C	Conventional	Conventional	1.000	0.000
PN-C	Conventional	Conventional	1.000	0.000
MH-C	Conventional	Conventional	1.000	0.000

Table S18. Confusion matrix for the cross-validation results (Raman data – red grape pulp extracts).

From / to	Conventional	Organic	Total	% correct
Conventional	4	0	4	100.00
Organic	0	4	4	100.00
Total	4	4	8	100.00

References

35. Brereton, R.G. *Chemometrics: Data Analysis for the Laboratory and Chemical Plant*; John Wiley & Sons, Ltd.: Chichester, UK, 2003; pp. 183-269, doi:10.1002/0470863242.
38. Radulescu, C.; Olteanu, R.L.; Stihi, C.; Florescu, M.; Stirbescu, R.M.; Stanescu, S.G.; Nicolescu, C.M.; Bumbac, M. Chemometrics-based vibrational spectroscopy for *Juglandis semen* extracts investigation. *J. Chemom.* **2020**, *34*, e3234, doi:10.1002/cem.3234.
39. Gautam, R.; Vanga, S.; Ariese, F.; Umapathy, S. Review of multidimensional data processing approaches for Raman and infrared spectroscopy. *EPJ Tech. Instrum.* **2015**, *2*, 1-38, doi:10.1140/epjti/s40485-015-0018-6.

41. Lasch, P. Spectral pre-processing for biomedical vibrational spectroscopy and microspectroscopic imaging. *Chemom. Intell. Lab. Syst.* **2012**, *117*, 100-114, doi:10.1016/j.chemolab.2012.03.011.
42. Biancolillo, A.; Marini, F. Chemometric Methods for Spectroscopy-Based Pharmaceutical Analysis. *Front. Chem.* **2018**, *6*, 576, doi:10.3389/fchem.2018.00576.
43. Smith, B.C. Fundamentals of Fourier Transform Infrared Spectroscopy, 2nd ed.; Taylor & Francis Group, CRC Press: Boca Raton, FL, USA, 2011, doi:10.1201/b10777.
44. Shen, X.; Xu, L.; Ye, S.; Hu, R.; Jin, L.; Xu, H.; Liu, W. Automatic baseline correction method for the open-path Fourier transform infrared spectra by using simple iterative averaging. Opt. Express. 2018, 26, A609–A614, doi:10.1364/OE.26.00A609.
45. Heraud, P.; Wood, B.R.; Beardall, J.; McNaughton, D. Effects of pre-processing of Raman spectra on in vivo classification of nutrient status of microalgal cells. J. Chemom. 2020, 20, 193–197, doi:10.1002/cem.990.
46. Kohler, A.; Kristian, A.N.; Martens, H. Chemometrics in Biospectroscopy. In Handbook of Vibrational Spectroscopy, 1st ed.; John Wiley & Sons, Ltd.: Hoboken, NJ, USA, 2010, doi:10.1002/0470027320.s8937.
47. Mazet, V.; Carteret, C.; Brie, D.; Idier, J.; Humbert, B. Background removal from spectra by designing and minimising a non-quadratic cost function. Chemom. Intell. Lab. Syst. 2005, 76, 121–133, doi:10.1016/j.chemolab.2004.10.003.
48. Randolph, T.W. Scale-based normalization of spectral data. Cancer Biomark. 2006, 2, 135–144, doi:10.3233/cbm-2006-23-405.
49. Sakia, R.M. The Box-Cox transformation technique: A review. J. R. Stat. Soc. Ser. D 1992, 41, 169–178, doi:10.2307/2348250.
50. Laakso, O.; Haapala, M.; Jaakkola, P.; Laaksonen, R.; Nieminen, J.; Pettersson, M.; Rasanen, M.; Himberg, J.J. The use of low-resolution FT-IR spectrometry for the analysis of alcohols in breath. J. Anal. Toxicol. 2000, 24, 250–256, doi:10.1093/jat/24.4.250.
51. Rayat, A.; Amirshahi, S.A.; Agahian, F. Compression of spectral data using Box-Cox transformation. Color Res. Appl. 2014, 39, 136–142, doi:10.1002/col.21771.
52. Brereton, R.G. Pattern Recognition. In Applied Chemometrics for Scientists; John Wiley & Sons, Ltd.: Hoboken, NJ, USA, 2007; pp. 145–191, doi:10.1002/9780470057780.
53. Bro, R.; Smilde, A.K. Principal component analysis. Anal. Methods 2014, 6, 2812–2831, doi:10.1039/c3ay41907j.
54. Buruleanu, L.C.; Radulescu, C.; Georgescu, A.A.; Danet, F.A.; Olteanu, R.L.; Nicolescu, C.M.; Dulama, I.D. Statistical Characterization of the Phytochemical Characteristics of Edible Mushroom Extracts. Anal. Lett. 2018, 51, 1039–1059, doi:10.1080/00032719.2017.1366499.
55. Jolliffe, I.T. Principal Component Analysis, 2nd ed.; Springer Series in Statistics; Springer: New York, NY, USA, 2002; pp. 78–110, doi:10.1007/b98835.
56. Greenacre, M. Power transformations in correspondence analysis. Comput. Stat. Data Anal. 2009, 53, 3107–3116, doi:10.2139/ssrn.1012787.
57. Lam, C. Visualizing Categorical Data: An Introduction to Correspondence Analysis for Technical Communication Researchers. In Proceedings of the 2014 IEEE International Professional on Communication Conference (IPCC), Pittsburgh, PA, USA, 13–15 October. 2014; pp. 1–7, doi:10.1109/IPCC.2014.7020345.
58. Everitt, B.S.; Dunn, G. Applied Multivariate Data Analysis, 2nd ed.; John Wiley & Sons Ltd.: Hoboken, NJ, USA, 2010; pp. 125–158.
59. Kamil, M.; Asri, M.N.; Desa, W.N.; Ismail, D. Fourier transform infrared (FTIR) spectroscopy and principal component analysis (PCA) of unbranded black ballpoint pen inks. Malays. J. Forensic Sci. 2015, 6, 48–53, doi:10.12816/0017699.
60. Ranganathan, P.; Pramesh, C.S.; Aggarwal, R. Common pitfalls in statistical analysis: Logistic regression. Perspect. Clin. Res. 2017, 8, 148–151, doi:10.4103/picr.PICR_87_17.
61. Yendle, P.W.; MacFie, H.J.H. Discriminant principal components analysis. J. Chemom. 1989, 3, 589–600, doi:10.1002/cem.1180030407.
62. Kemsley, E.K. Discriminant analysis of high-dimensional data: A comparison of principal components analysis and partial least squares data reduction methods. Chemom. Intell. Lab. Syst. 1996, 33, 47–61, doi:10.1016/0169-7439(95)00090-9.
